# A Novel Device for Intraoperative Measurement of Stem Anteversion Angle in Total Hip Arthroplasty

**DOI:** 10.1016/j.artd.2024.101458

**Published:** 2024-07-20

**Authors:** Kentaro Iwakiri, Yoichi Ohta, Shuhei Ueno, Yukihide Minoda, Akio Kobayashi, Hiroaki Nakamura

**Affiliations:** a Department of Orthopaedic Surgery, Shiraniwa Hospital Joint Arthroplasty Center, Nara, Japan; b Department of Orthopaedic Surgery, Osaka Metropolitan University Graduate School of Medicine, Osaka, Japan

**Keywords:** Stem anteversion, Total hip arthroplasty, Intraoperative measurement, Stem anteversion angle, Three-dimensional measurements

## Abstract

**Background:**

Stem anteversion plays a crucial role in mitigating postoperative complications in total hip arthroplasty (THA). The application of the combined-anteversion theory in THA necessitates the intraoperative measurement of the stem anteversion angle (SAA). However, estimating SAA intraoperatively poses challenges for surgeons lacking a computer-assisted navigation system. In this study, we assessed the precision of intraoperative SAA measurements using a recently developed device, comparing them with 3-dimensional measurements obtained from postoperative computed tomography.

**Methods:**

We examined 127 hips in 127 patients who underwent unilateral THA at our institution. Employing our newly constructed device, attachable to rasping broach handles, we measured the SAA intraoperatively. This process involved incorporating the correction angle derived from the preoperative epicondylar view. We then compared the postoperative SAA with the intraoperative measurements, both with and without the correction angle, to ascertain the device's utility.

**Results:**

The device yielded an intraoperative SAA measurement of 17.93 ± 7.53°. In contrast, the true SAA measured on postoperative computed tomography was 26.40 ± 9.73°. The discrepancy between intraoperative and true SAA was 8.94 ± 5.44° (without the correction angle) and 4.93 ± 3.85° (with the correction angle). Accuracy within a discrepancy of <5° was achieved in 77 cases (60.6%), and <10° was achieved in 113 cases (89.0%). The accuracy remained consistent regardless of the stem-placement angle (varus/valgus, or flexion/extension) or the presence of ipsilateral knee osteoarthritis.

**Conclusions:**

The SAA-measuring device, attachable to various rasping handles, proves useful for straightforward, cost-effective, and noninvasive intraoperative SAA measurement during THA.

## Introduction

Precise implant placement is pivotal for achieving favorable outcomes in total hip arthroplasty (THA). Improper implant positioning elevates the risk of postoperative complications, including dislocation, impingement, restricted range of motion, accelerated polyethylene wear, and aseptic loosening [[1], [2], [3], [4], [5]]. Consequently, the accuracy of implant placement stands as a critical factor in averting these complications.

Numerous studies have elucidated optimal cup placement angles. In 1978, Lewinneck et al. initially defined the safe zone for the cup, recommending an inclination of 40 ± 10° and an anteversion of 15 ± 10°—a benchmark still adhered to by some surgeons [1]. In clinical practice, various tools such as computer-assisted navigation systems [5,6], accelerometer-based portable navigation [7], robotic-assisted systems [8], and mechanical navigators [[9], [10], [11], [12], [13]] have proven efficacious in achieving precise cup placement. However, literature on the ideal stem anteversion angle (SAA) on the femoral side remains scarce, with reports indicating wide variability in the anteversion angle of femoral stems.

The concept of combined anteversion (sum of cup anteversion and femoral anteversion) in THA was introduced by Ranawat and Maynard in 1991 [14]. Studies by Jolles et al. and Widmer and Zurfluh underscored the significance of stem anteversion, akin to cup anteversion, in minimizing postoperative complications in THA [15,16]. Nevertheless, estimating SAA intraoperatively proves challenging for surgeons without access to expensive and intricate computer-assisted navigation systems. Our prior work introduced a device facilitating accurate, easy, and stable cup placement angle setting in THA [10,17]. In this study, we present a novel device for measuring SAA intraoperatively and assess its accuracy by comparing measurements with 3-dimensional evaluations obtained from postoperative computed tomography (CT) and templating software.

## Material and methods

### Ethics statements

The research protocol received approval from our institutional review board. Prior to participation, all patients provided written informed consent. The trial was registered with the University Hospital Medical Information Network under the registration number (UMIN000035913).

## Study population

The study encompassed 127 patients who underwent primary THA at our hospital between February 1, 2019, and March 31, 2020. Among them, 116 were women, and 11 were men, all diagnosed with hip osteoarthritis (OA). Exclusion criteria included patients slated for simultaneous bilateral THA or revision THA. In addition, individuals with a history of prior surgery on the affected hip joint or knee on the affected side were excluded from the study (Table 1).

**Table 1:** Demographics of the 127 patients.

Variables	Value
Age at surgery (years)^a^	68.2 ± 10.3 (41∼93)
Gender (male/female)	11/116
Body mass index (kg/m^2^)^a^	
Male	25.2 ± 2.1 (22.9∼29.6)
Female	24.1 ± 4.0 (15.6∼35.0)
Preoperative diagnosis (number of hips)	
Osteoarthritis	121
Osteonecrosis	6
Osteoarthritis of the knee at the affected side	
KL 1	68
KL 2	38
KL 3	17
KL 4	4
Implant	
Taperloc	62
Actis	26
AccoladeII	25
Entrada	14
Postoperative stem placement angle from 3D measurements (°)^a^	
Anteversion	26.40 ± 9.73
Varus	1.89 ± 1.84
Flexion	3.24 ± 1.67

a Values expressed as mean ± standard deviation, with range in parentheses.

All surgical procedures were conducted using the anterolateral intermuscular approach, with patients positioned laterally. A single surgeon (K.I.) performed all operations. The stems employed were Taperloc (ZimmerBiomet, Warsaw, IN) in 62 patients, Actis (DePuySynthes, Warsaw, IN) in 26, AccoladeII (Stryker, Mahwah, NJ) in 25, and Entrada (OrthoDevelopment, Salt Lake, UT) in 14. The novel device was utilized to measure the stem's anteversion angle intraoperatively in all patients.

**Figure 1:**
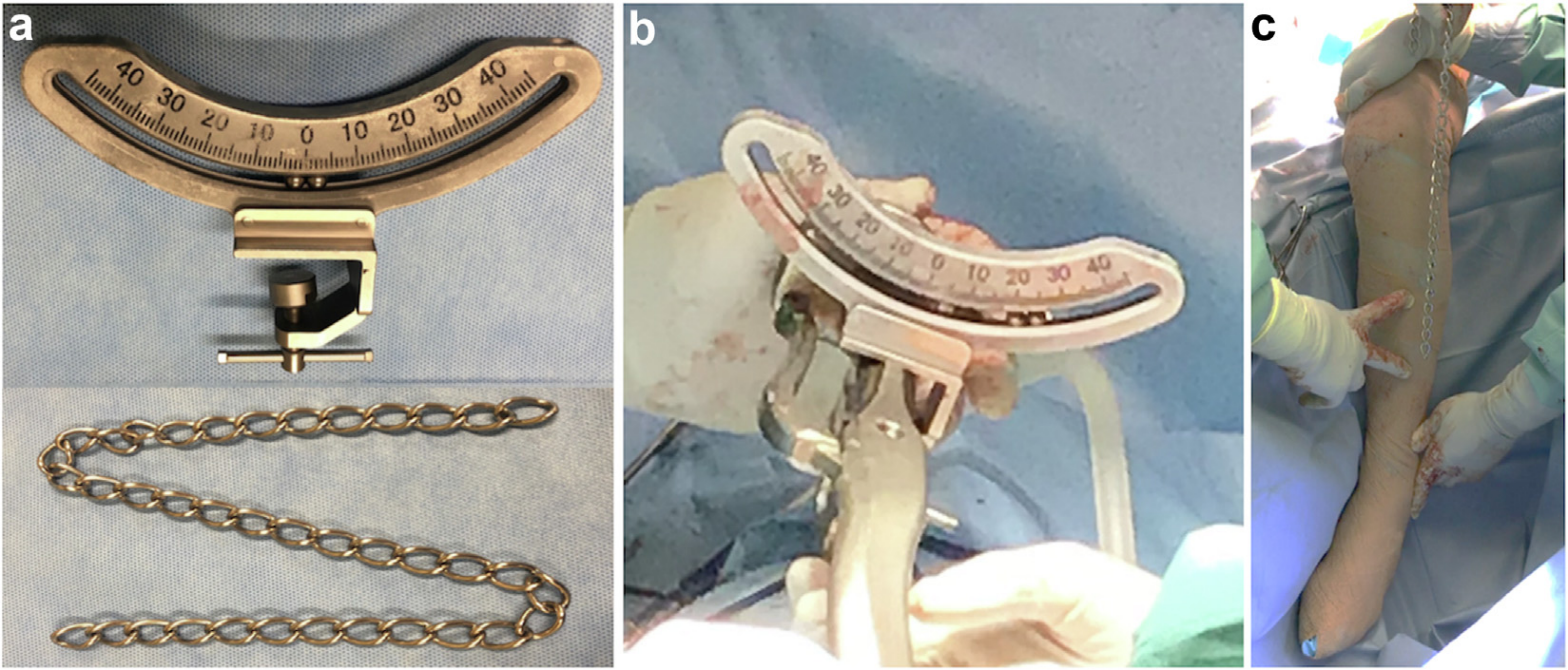
(a) The device and the weighted chain. The stem anteversion angle (SAA)-measuring device features an in-built marker consistently oriented toward the direction of the center of gravity. (b) Attachment of rasping broach handles: The device can attach to various types of rasping broach handles. (c) Intraoperative SAA measurements: During surgery, the intraoperative SAA is indicated by the device marker when the surgeon's assistant aligns the lower leg so that the weighted chain (representing the direction of the center of gravity) and the lower leg (marked by the tibial ridge line with fingers pointed out) are parallel to each other.

Our newly developed device attaches to various types of rasping broach handles, enabling the measurement of the twist angle of the rasp handle concerning the direction of gravity (Fig. 1a and b). Generally, SAA is defined as the anteverted angle of the stem from the plane (table-top plane), incorporating the posterior contact points of the medial and lateral posterior condyles of the femur and the posterior contact point of the greater trochanter [18]. Intraoperatively, the posterior contact points of both medial and lateral posterior condyles cannot be visualized or accessed; hence, the SAA cannot be measured using the axis of the posterior condyle of the femur as a reference.

**Figure 2:**
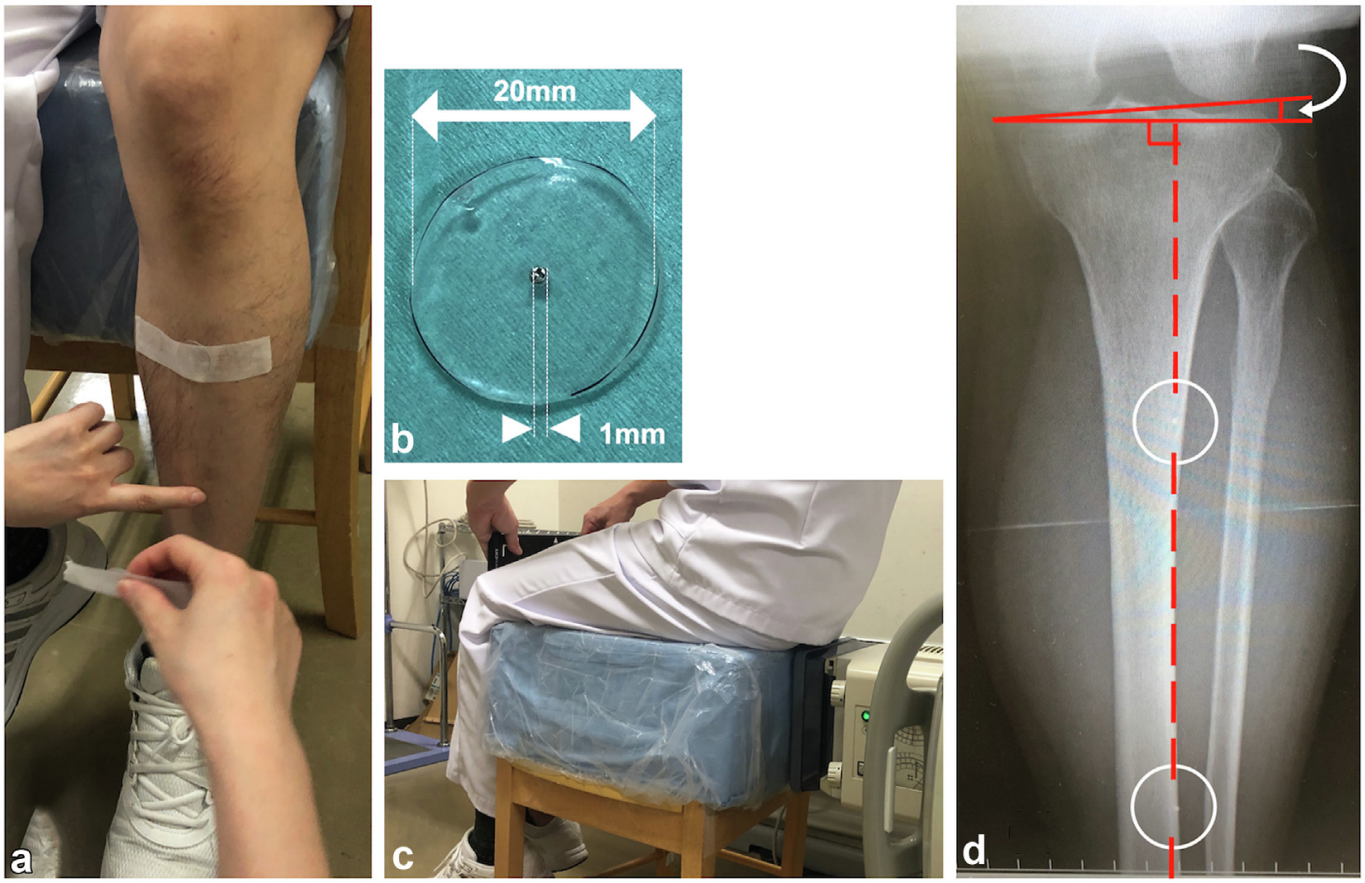
The procedure for capturing the preoperative epicondylar view. (a) Placement of metal markers: Two metal markers are affixed, with one positioned 5 cm above and the other 5 cm below the center of the tibial ridge. (b) Composition of the 1-mm metal marker: A 1-mm metal marker is centrally located on a 20-mm acrylic plate. (c) Patient positioning for Xp imaging: The patient is positioned to facilitate the acquisition of the Xp image. (d) Correction angle indication: The white arrow highlights the correction angle, formed by the perpendicular line of the tibial ridge (lower leg) axis and the posterior condylar axis. Two metal markers are situated within the solid line of the white circle. The red dotted line represents the tibial ridge line.

To address this challenge, a single Xp image of the epicondyle (Kanekasu view) was captured preoperatively for each patient, with metal markers affixed 5 cm above and below the center of the tibial ridge [19]. The correction angle, formed by the perpendicular line of the tibial ridge line and the posterior condylar axis of the femur in the image, was then measured (Fig. 2). This measurement enabled the identification of the posterior condylar axis from the direction of the palpable tibial ridge line intraoperatively. Previous studies reported that the axis connecting the 2 points, 5 cm above and below the center of the tibial ridge, is parallel to the tibial axis in 97% of individuals [20].

## Device operation and measurements

**Figure 3:**
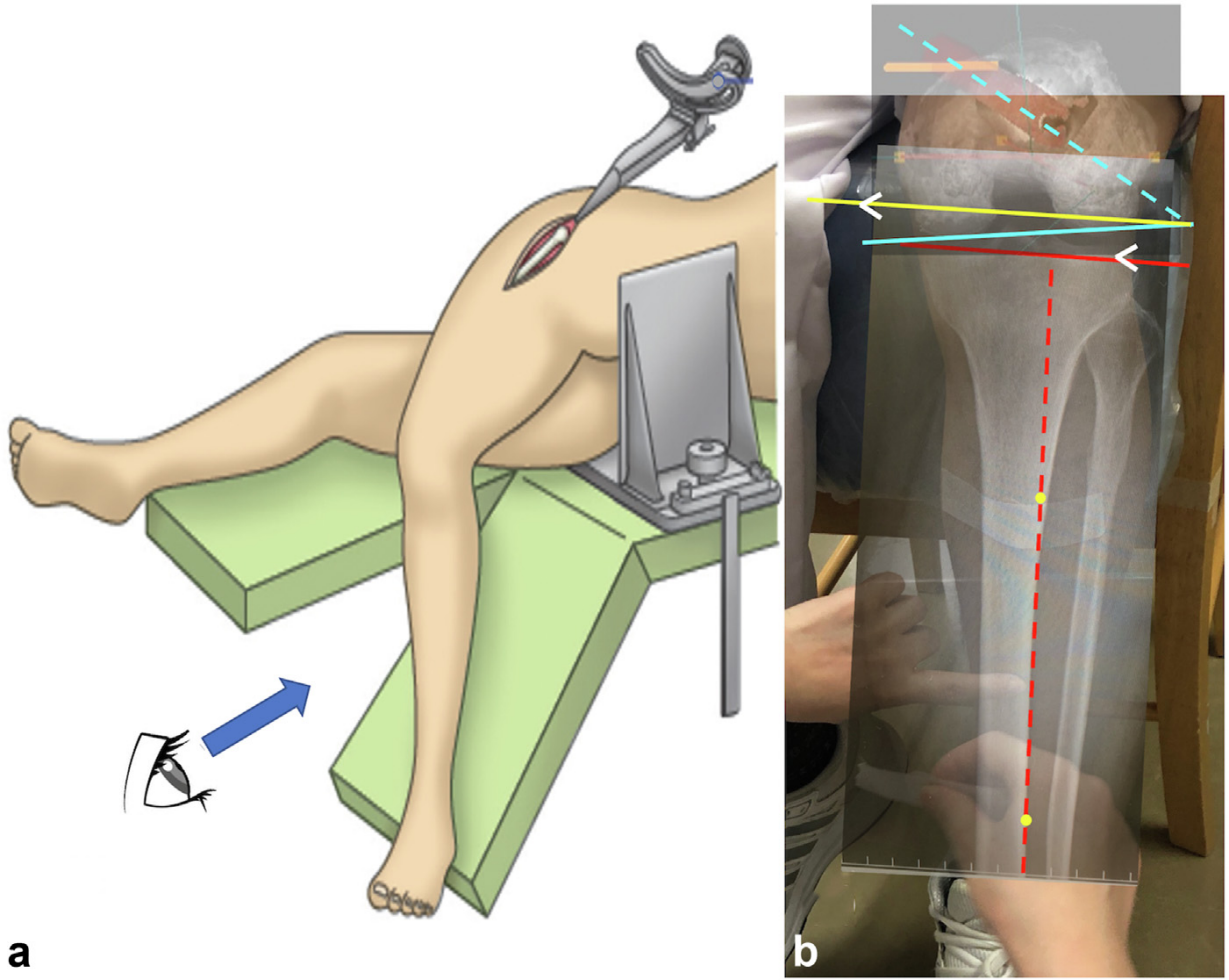
A schematic diagram illustrating the anteversion angle is presented. (a) Visualization of the entire lower limb during surgery. The device is attached to the holder of the final rasp, and the assistant maintains the lower leg in a vertical direction, as shown in Figure 1c. (b) We illustrated the relationship between intraoperative SAA (stem anteversion angle), correction angle, and anteversion angle in an artwork viewed from the anterior aspect of the lower leg. Intraoperative SAA is measured between the blue dotted line and the yellow line (perpendicular to the red dotted line). The correction angle is measured between the PCA (posterior condylar axis) line (blue solid line) and the red solid line (perpendicular to the tibial ridge line) and between the PCA line (blue solid line) and the yellow line (parallel to the red solid line). The corrected SAA (stem anteversion angle; Fig. 4c) is calculated as the angle between the blue lines, which is equal to the sum of the intraoperative SAA and the correction angle.

Following the intraoperative insertion of the final rasping broach, the surgeon effortlessly affixes the device to the rasping handle (Video 1). To align the tibial ridge line of the lower leg with the center of gravity, the surgeon's assistant holds the lower leg in a manner ensuring parallelism between the weighted chain and the tibial ridge line (Fig. 1c, Video 2). The tibial ridge line remains palpable during surgery. The device features an in-built marker consistently oriented toward the direction of the center of gravity (Fig. 1a, Video 3). The corrected SAA is determined by adding the correction angle (the angle formed by the perpendicular line of the tibial ridge line and the posterior condylar axis of the femur in the preoperative epicondylar view) to the intraoperative SAA indicated by the device marker, aligning the lower leg (tibial ridge) with the direction of the center of gravity while the assistant holds it. The comprehensive view of the lower limb during surgery, along with the relationship between intraoperative SAA, the correction angle, and the corrected SAA, is illustrated in the artwork (Fig. 3a and b).

**Figure 4:**
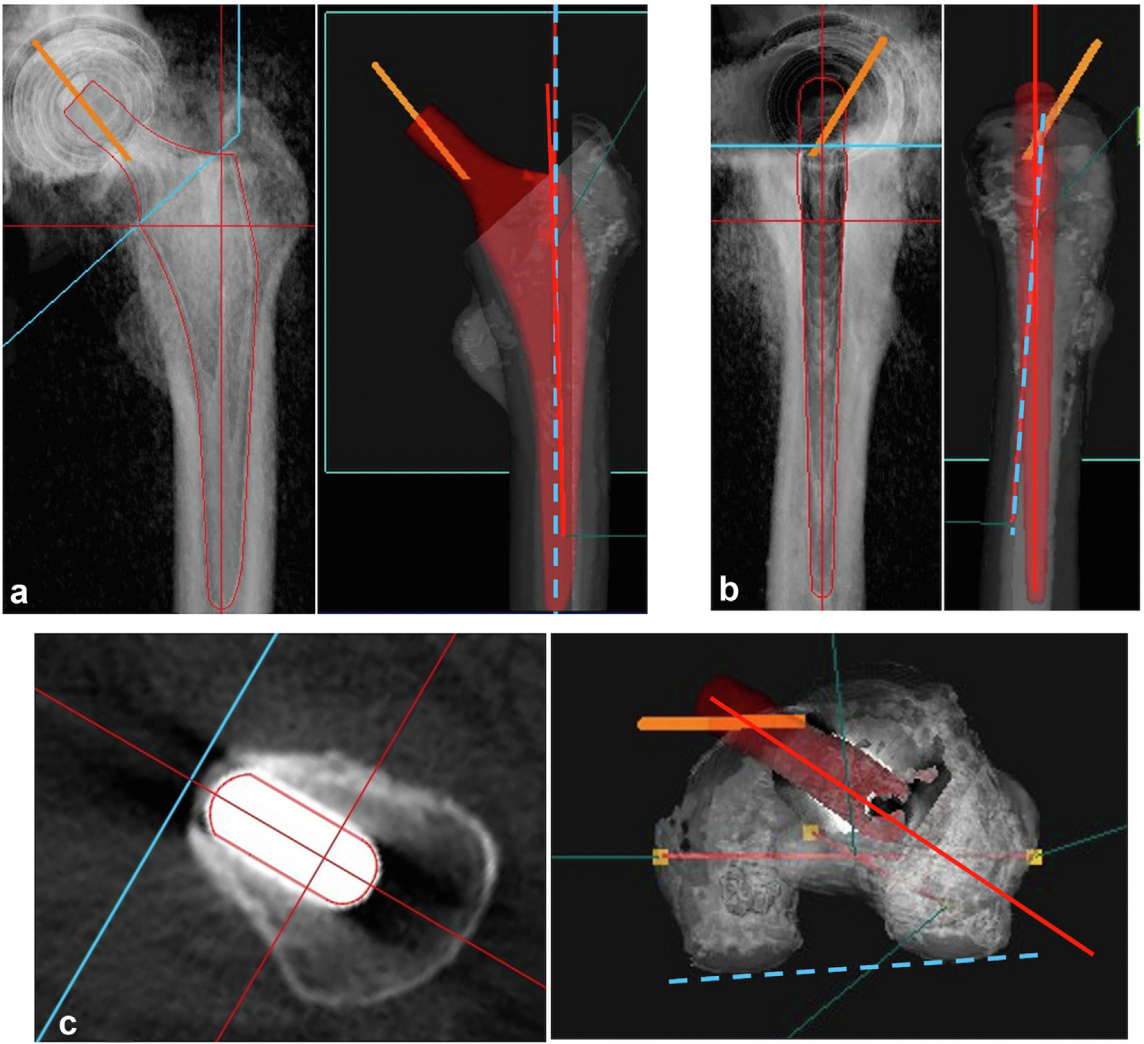
Stem alignment determination using a 3D templating software program and computed tomography. (a) Anteroposterior (AP) view for varus angle: The AP view is utilized for assessing the varus angle. (b) Lateral view for flexion angle: The lateral view is employed to evaluate the flexion angle. The blue dotted line represents the femoral axis, while the red solid line depicts the stem axis. The varus or flexion angle is derived from the angle between these 2 lines. (c) Axial view: In the axial view, the blue dotted line corresponds to the posterior condylar line (tabletop plane), and the red solid line represents the stem neck axis. The anteversion angle is calculated based on the angle between these 2 lines.

At the 3-month postoperative mark, the true SAA was measured using CT with a 3-dimensional templating software program (ZedHip; LEXI, Tokyo, Japan; Fig. 4). The femoral coordinate systems were established on the surface models based on the following definitions: the femoral axis as the line passing through the center of the third, fourth, and fifth portions in 14 equally divided sections of the femur. This axis was reflected by the stem in the coronal and sagittal planes. Varus or valgus was defined as the angle between the femoral axis and the stem axis, each reflected on the coronal plane of the stem, while flexion or extension was defined as the angle between the femoral axis and the stem axis, each reflected on the sagittal plane of the stem. Varus or flexion was denoted as the varus or flexion angle of the stem axis against the femoral axis on the corresponding defined coordinate plane. For anteversion or retroversion, the table-top plane was utilized, as previously described [18].

The accuracy of the device was validated by comparing (A) the intraoperative SAA indicated by the device marker (Fig. 3b), (B) the corrected SAA obtained by adding the intraoperative SAA to the correction angle (Fig. 3b), and (C) the true SAA measured on postoperative CT (Fig. 4c). Subsequently, we analyzed the correlation between (A) and (C), or between (B) and (C). In addition, we examined whether the discrepancy in accuracy was influenced by the stem placement angle (varus/valgus, flexion/extension), the grade of knee joint OA on the affected side, and the type of stem implant.

## Statistical analysis

Statistical analyses were conducted using SPSS version 26.0 (IBM Corp, Armonk, NY). Pearson’s correlation coefficient was employed to compare (A) to (C), or (B) to (C), as previously defined. The absolute values of the correlation coefficients were computed, with values <0.40 characterized as a weak correlation, values ranging from 0.40 to 0.70 defined as moderate, and values ≥0.70 considered a strong correlation. The Student *t*-test was executed to identify differences in the absolute value of the discrepancy between (A) and (C) or between (B) and (C) within the groups (varus >3 vs varus ≤3, including valgus) or the groups (flexion >3 vs flexion ≤3, including extension). One-way analysis of variance (ANOVA) was performed to identify differences in the absolute value of the discrepancy between (A) and (C) or between (B) and (C) within the Kellgren and Lawrence (KL) classification groups or the groups categorized by each type of stem [21]. The level of significance was set at *P* = .05.

## Results

The intraoperative SAA measured by the device was 17.93 ± 7.53° (range: 1°-44°). In contrast, the true SAA measured from postoperative CT was 26.40 ± 9.73° (range: 0.18°-45.67°). Without considering the correction angle between the perpendicular line of the tibial ridge line and the posterior condylar axis of the femur (measured using an Xp epicondylar view), the absolute value of the discrepancy between the intraoperative SAA and the true SAA was 8.94 ± 5.44° (range: 0°-24.67°), demonstrating a strong positive correlation between these 2 values (r = 0.775). Among the 127 cases, 36 (28.3%) had a discrepancy within 5° from the true SAA, and 81 (63.8%) had a discrepancy within 10° (Table 2).

**Table 2:** Discrepancy between intraoperative SAA with or without adding the correction angle and the true SAA measured by 3D measurement.

Without adding the correction angle	Number (%)	Mean ± SD	*P* value^a^	r^b^	With adding the correction angle	Number (%)	Mean ± SD	*P* value^a^	r^b^
					Correction angle (°)	127	6.94 ± 3.13		
(A) intraoperative SAA indicated by the device (°)	127	17.93 ± 7.53	<.001	0.775	(B) corrected SAA with adding the correction angle (°)	127	24.76 ± 8.24	<.001	0.786
(C) True SAA measured by 3D measurements (°)	127	26.40 ± 9.73			(C) true SAA measured by 3D measurements (°)	127	26.40 ± 9.73		
Discrepancy between (A) and (C) (absolute value) (°)	127	8.94 ± 5.44			Discrepancy between (B) and (C) (absolute value) (°)	127	4.93 ± 3.85		
Range					Range				
−15< ≤−10	0 (0%)				−15< ≤-10	4 (3.1%)			
−10< ≤−5	3 (2.4%)				−10< ≤−5	10 (7.9%)			
−5< ≤5	36 (28.3%)				−5< ≤5	77 (60.6%)			
5< ≤10	42 (33.1%)				5< ≤10	26 (20.5%)			
10< ≤15	27 (21.3%)				10< ≤15	10 (7.9%)			
15< ≤20	13 (10.2%)				15< ≤20	1 (0.8%)			
20< ≤25	6 (4.7%)				20< ≤25	0 (0%)			

SAA, stem anteversion angle; SD, standard deviation; 3D, three-dimensional.

a *P* value from pearson's correlation coefficient.

b Value of the correlation coefficients.

**Figure 5:**
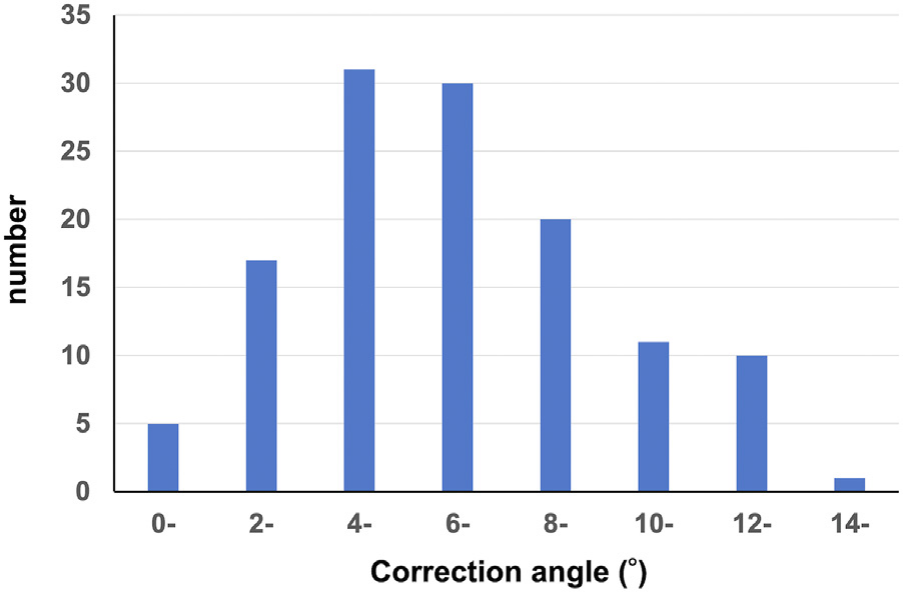
Correction angle distribution in the preoperative epicondylar view: The correction angle, established by the perpendicular alignment of the tibial ridge line and the posterior condylar axis of the femur in the preoperative epicondylar view, exhibited a consistently positive value in all cases. This positive value indicates a larger lateral space on the femoral-tibial articular surface than the medial space.

On the other hand, when considering the correction angle, the corrected SAA, obtained by adding the intraoperative SAA with the correction angle, was 24.76 ± 8.24° (range: 4.64°-44.50°). The absolute value of the discrepancy between the corrected SAA and the true SAA was 4.93 ± 3.85° (range: 0.01°-18.36°), indicating a strong positive correlation between these 2 values (r = 0.786). Notably, 77 of the 127 cases (60.6%) had a discrepancy within 5° from the true SAA, and 113 cases (89.0%) had a discrepancy within 10° (Table 2). The incorporation of the correction angle (formed by the perpendicular line of the tibial ridge and the posterior condylar axis of the femur) effectively reduced the discrepancy. The correction angle was measured at 6.94 ± 3.13° (range: 0.39°-14.25°), with all cases exhibiting positive values, signifying a larger lateral space on the femoral-tibial articular surface than on the medial space (Fig. 5).

Based on the KL classification of OA knee, 68 patients were classified as KL-1, 38 patients as KL-2, 17 patients as KL-3, and 4 patients as KL-4 [21]. Moreover, there was no discernible association between the KL classification and the intraoperative SAA, the corrected SAA, the true SAA, the correction angle, or the discrepancies from the true SAA (Table 3).

**Table 3:** Comparison of each value and discrepancies between each KL group.

	Total	KL 1	KL 2	KL 3	KL 4	*P* value
Number	127	68	38	17	4	
(A) Intraoperative SAA indicated by the device^a^ (°)	17.93 ± 7.53	17.82 ± 7.63	17.85 ± 6.93	16.21 ± 8.79	19.38 ± 7.15	.096^b^
Correction angle^a^ (°)	6.94 ± 3.13	6.74 ± 3.06	6.79 ± 3.09	7.42 ± 3.30	9.56 ± 3.85	.314^b^
(B) Corrected SAA with adding the correction angle^a^ (°)	24.76 ± 8.24	24.36 ± 8.06	25.54 ± 7.75	23.63 ± 10.33	28.94 ± 7.00	.193^b^
(C) True SAA measured by 3D measurements^a^ (°)	26.40 ± 9.73	26.43 ± 9.37	28.01 ± 9.82	22.94 ± 11.60	25.47 ± 2.75	.281^b^
Discrepancy between (A) and (C) (absolute value)^a^ (°)	8.94 ± 5.44	8.75 ± 5.66	9.66 ± 5.37	8.11 ± 5.32	8.98 ± 2.83	.460^b^
Discrepancy between (B) and (C) (absolute value)^a^ (°)	4.93 ± 3.85	4.71 ± 3.53	4.76 ± 4.13	6.23 ± 4.12	4.82 ± 5.58	.091^b^

KL, Kellgren and Lawrence; SAA, stem anteversion angle; 3D, three-dimensional.

a The values are given as the mean and standard deviation.

b The *P* values were determined with the one-way ANOVA test.

No correlation was observed between the varus and valgus stem placement angles and the discrepancies from the true SAA. Similarly, there was no correlation between the flexion placement angle and the discrepancy. No significant difference was found between cases of varus or valgus insertion over 3° and cases of varus or valgus insertion less than 3°, as well as between flexion insertions over 3° and flexion insertions of less than 3° (Table 4, Table 5). In addition, no association was identified between the types of stem implant and the intraoperative SAA, the corrected SAA, the true SAA, the correction angle, or the discrepancies from the true SAA (Table 6).

**Table 4:** Comparison of the discrepancies between cases of varus insertion more than 3° and cases of varus or valgus insertion less than 3°.

	Total	Neutral	Varus	Valgus	*P* value
		Varus/Valgus ≤3	Varus >3	Valgus >3	
Number	127	93	34	0	
Varus/Valgus angle^a^ (°)	1.89 ± 1.84	1.05 ± 1.30	4.21 ± 0.85	-	.018^b^
Discrepancy between (A) and (C) (absolute value)^a^ (°)	8.94 ± 5.44	8.27 ± 5.31	10.79 ± 5.44	-	.238^b^
Discrepancy between (B) and (C) (absolute value)^a^ (°)	4.93 ± 3.85	4.52 ± 3.76	6.05 ± 3.92	-	.404^b^

(A) intraoperative SAA indicated by the device (°). (B) Corrected SAA with adding the correction angle (°). (C) True SAA measured by 3D measurements (°).

a The values are given as the mean and standard deviation.

b The *P* value were determined with the Student *t*-test.

**Table 5:** Comparison of the discrepancies between cases of flexed insertion more than 3° and cases of flexed or extended insertion less than 3°.

	Total	Neutral	Varus	Valgus	*P* value
		Flexion/Extension ≤ 3	Flexion >3	Extension >3	
Number	127	59	68	0	
Flexion/Extention angle^a^ (°)	3.24 ± 1.67	1.81 ± 0.92	4.48 ± 1.08	-	.013^b^
Discrepancy between (A) and (C) (absolute value)^a^ (°)	8.94 ± 5.44	9.40 ± 6.03	8.54 ± 4.87	-	.532^b^
Discrepancy between (B) and (C) (absolute value)^a^ (°)	4.93 ± 3.85	5.30 ± 4.17	4.60 ± 3.54	-	.628^b^

(A) intraoperative SAA indicated by the device (°). (B) Corrected SAA with adding the correction angle (°). (C) True SAA measured by 3D measurements (°).

a The values are given as the mean and standard deviation.

b The *P* values were determined with the Student *t*-test.

**Table 6:** Comparison of each value and discrepancies between each type of stem.

	Total	Taperloc	Actis	AccoladeII	Entrada	*P* value
Number	127	62	26	25	14	
(A) Intraoperative SAA indicated by the device^a^ (°)	17.93 ± 7.53	19.14 ± 7.07	18.87 ± 8.85	16.30 ± 6.66	13.79 ± 7.10	.058^b^
Correction angle^a^ (°)	6.94 ± 3.13	7.42 ± 3.31	6.93 ± 3.00	5.57 ± 2.56	7.31 ± 3.10	.095^b^
(B) Corrected SAA with adding the correction angle^a^ (°)	24.76 ± 8.24	26.31 ± 7.60	25.80 ± 9.64	21.87 ± 6.30	21.10 ± 9.55	.103^b^
(C) True SAA measured by 3D measurements^a^ (°)	26.40 ± 9.73	28.41 ± 8.71	26.83 ± 8.65	22.30 ± 10.20	24.07 ± 12.95	.188^b^
Discrepancy between (A) and (C) (absolute value)^a^ (°)	8.94 ± 5.44	9.51 ± 5.16	8.52 ± 5.17	6.81 ± 4.69	11.03 ± 7.36	.089^b^
Discrepancy between (B) and (C) (absolute value)^a^ (°)	4.93 ± 3.85	4.68 ± 3.71	5.26 ± 3.47	4.81 ± 4.33	5.63 ± 4.49	.516^b^

Taperloc (ZimmerBiomet, Warsaw, IN), Actis (DePuySynthes, Warsaw, IN), AccoladeII (Stryker, Mahwah, NJ), Entrada (OrthoDevelopment, Salt Lake, UT).

a The values are given as the mean and standard deviation.

b The *P* values were determined with the one-way ANOVA test.

## Discussion

The outcomes of this investigation underscore the feasibility of seamlessly measuring the SAA intraoperatively using the devised device. This capability, previously absent without the use of an elaborate and expensive navigation system, exhibited an average absolute value discrepancy of 8.94°. However, when rectified by incorporating Xp imaging of the epicondylar view before surgery and measuring the correction angle, formed by the perpendicular line of the tibial ridge and the posterior condylar axis of the femur, the corrected SAA demonstrated a high accuracy with an average absolute value discrepancy of 4.93°. Impressively, 60.6% of cases revealed a discrepancy within 5° of the true anteversion value, and 89.0% exhibited a discrepancy within 10°. In the previous study by Kitada et al., utilizing CT-based navigation, the accuracy of stem anteversion was reported to be within 5° in 77% of cases and within 10° in 97% of cases [6]. While the accuracy of our device usage is inferior to CT-based navigation, it is noteworthy that high precision is achieved without the need for invasive procedures such as pin insertion into the femur, as seen in CT-based navigation. In addition, the device showcased versatility by effortlessly attaching to various models of rasping broach handles, maintaining consistent accuracy. Notably, the accuracy remained unaffected by stem placement angles (varus/valgus or flexion/extension) or the presence of knee OA on the affected side.

Ranawat and Maynard initially introduced the concept of combined anteversion, emphasizing the summation of cup anteversion and femoral anteversion in THA [14]. Jolles et al. highlighted that a deviation in combined anteversion, specifically ranging from 40° to 60°, correlated with an increased dislocation rate [15]. Widmer and Zurfluh proposed the ideal value as the sum of stem anteversion and 0.7 times cup anteversion, totaling 37.3 in a 3-dimensional model analysis [16]. This underscores the significance of stem anteversion, akin to cup anteversion, in mitigating postoperative complications of THA. While reports indicate the possibility of accurately setting cup anteversion at a low cost using accelerometer-based portable navigation, the accuracy of stem anteversion determination during surgery, apart from computer-assisted navigation, remains limited [7,[9], [10], [11], [12], [13]]. Existing alternatives, like the gravity guide proposed by Fujihara et al., possess a reported accuracy of 4.6 ± 4.1 but entail the cumbersome attachment of a large device to the lower leg, making the procedure intricate and unsuitable for various stem types [22]. Moreover, the accuracy's reliability in cases of knee OA remains unknown.

Stem anteversion significantly influences THA outcomes. However, its adjustment during surgery is challenging due to patient-specific femoral anteversion, the level of femoral neck osteotomy, and the stem flexion/extension angle. Dorr et al. reported substantial variability in SAA when the stem is set without computer-assisted navigation [23]. Surgeon-estimated anteversion often results in a discrepancy of 10° or more, leading to varied outcomes [24]. Normal anteversion (10-15°) was present in 8.3% of patients, and the prevalence of abnormal stem antetorsion was 92% [25]. Intraoperative control of SAA differs between stem types, with tapered-wedge stems offering a larger adjustment range than fit-and-fill–type stems [26,27]. Apart from our innovative device, computer-assisted navigation remains the primary method for accurately measuring stem anteversion during surgery. While Kitada et al. reported high correlation percentages between navigation-indicated SAA and postoperative CT measurements, the widespread availability of navigation systems to surgeons remains limited [6]. Furthermore, even with the use of CT-based navigation, it has been reported that the accuracy of stem insertion angles is more variable in terms of anteversion/retroversion than in terms of varus/valgus and flexion/extension [6]. The ability to intraoperatively grasp the most variable angle, anteversion, without the need for expensive tools such as navigation or robotics, and without the invasive step of inserting pins into the femur, can be an extremely useful tool for many surgeons.

This study possesses notable strengths. The scrutiny of knee OA on the affected side is a critical aspect, as many THA patients present with concomitant knee OA. Regardless of the presence of knee OA, our device demonstrated high accuracy. The attachment of 2 markers on the tibial ridge during Xp imaging enhanced accuracy given the reported alignment of the axis connecting these markers with the lower leg axis in 97% of cases. The palpability of the tibial ridge line intraoperatively further contributed to increased accuracy. In addition, postoperative CT evaluations, conducted with a 3-dimensional templating software program, ensured highly accurate measurements of stem anteversion, varus/valgus, and extension/flexion angles.

While acknowledging limitations, such as potential discrepancies in visually aligning the tibial ridge line and variations in final rasp and stem placements, our study substantiates the utility of the developed SAA measuring device. Although additional time and effort are required to capture a single preoperative x-ray image for the correction angle, the potential benefits may outweigh the invested resources. As this study is based on the results of the anterolateral approach, it is necessary to further investigate the accuracy of the device in other approaches in the future. This device emerges as a cost-effective and noninvasive alternative, offering precise measurements during THA, aligning with the combined-anteversion theory.

## Conclusions

The SAA-measuring device utilized in our study has been validated as a valuable tool for precise measurement of the SAA during THA. Its utility extends across various stem types, positioning it as a straightforward, cost-effective, intuitive, noninvasive, and practical alternative to computer-assisted navigation. This aligns with the principles of the combined-anteversion theory, underscoring its potential as a reliable instrument for enhancing the accuracy of THA procedures.

## Acknowledgments

The authors thank Yohei Ohyama, Shingo Maeda, Sho Masuda, and Takashi Fujii for the assistants of the orthopedic surgery in Shiraniwa Hospital.

## Funding

This research did not receive any specific grant from funding agencies in the public, commercial, or not-for-profit sectors.

## Conflicts of interest

The authors declare there are no conflicts of interest.

For full disclosure statements refer to https://doi.org/10.1016/j.artd.2024.101458.

## CRediT authorship contribution statement

**Kentaro Iwakiri:** Writing – original draft, Validation, Methodology, Data curation. **Yoichi Ohta:** Visualization, Software. **Shuhei Ueno:** Formal analysis, Data curation. **Yukihide Minoda:** Writing – review & editing, Visualization. **Akio Kobayashi:** Visualization. **Hiroaki Nakamura:** Supervision, Project administration, Methodology.

## Appendix A Supplementary Data

Conflict of Interest Statement for Kobayashi

Conflict of Interest Statement for Nakamura

Conflict of Interest Statement for Iwakiri

Conflict of Interest Statement for Ueno

Conflict of Interest Statement for Minoda

Conflict of Interest Statement for Ohta
