# Supplementary material for: A Novel Device for Intraoperative Measurement of Stem Anteversion Angle in Total Hip Arthroplasty
Source: Arthroplast Today. 2024 Jul 20;28:101458. doi: 10.1016/j.artd.2024.101458 (PMC11295631; doi:10.1016/j.artd.2024.101458)
Supplement: Conflict of Interest Statement for Kobayashi [file mmc1.pdf]

CONFLICT OF INTEREST STATEMENT

American Association of Hip and Knee Surgeons

(Adopted from the American Academy of Orthopaedic Surgeons disclosure statement)

The following form **must be filled out completely and submitted by each author (example, 6 authors, 6 forms). All items require a response. If there is no relevant disclosure for a given item, enter "*None*."**

Manuscript Title: Intraoperative measurement of stem anteversion angle in total hip arthroplasty: A novel device for simple and precise application

1. Royalties from a company or supplier (The following conflicts were disclosed)

   none

2. Speakers bureau/paid presentations for a company or supplier (The following conflicts were disclosed)

   none

3A. Paid employee for a company or supplier (The following conflicts were disclosed)

   none

3B. Paid consultant for a company or supplier (The following conflicts were disclosed)

   none

3C. Unpaid consultants for a company or supplier (The following conflicts were disclosed)

   none

4. Stock or stock options in a company or supplier (The following conflicts were disclosed)

   none

5. Research support from a company or supplier as a Principal Investigator (The following conflicts were disclosed)

   none

6. Other financial or material support from a company or supplier (The following conflicts were disclosed)

   none

7. Royalties, financial or material support from publishers (The following conflicts were disclosed)

   none

8. Medical/Orthopaedic publications editorial/governing board (The following conflicts were disclosed)

   none

9. Board member/committee appointments for a society (The following conflicts were disclosed)

   none

Each author must sign AND print or type his/her name, date and submit a separate form

In addition, one BLINDED Conflict of Interest form (no author names used) should be submitted per manuscript with all author disclosures.

Akio Kobayashi — Akio Kobayashi — 12 Jan 2024

Author Name (Print or Type) — Author Signature — Date
